# Why we should care about gas pockets in online adaptive MRgRT: a dosimetric evaluation

**DOI:** 10.3389/fonc.2023.1280836

**Published:** 2023-11-13

**Authors:** Matteo Nardini, Guenda Meffe, Matteo Galetto, Luca Boldrini, Giuditta Chiloiro, Angela Romano, Giulia Panza, Andrea Bevacqua, Gabriele Turco, Claudio Votta, Amedeo Capotosti, Roberto Moretti, Maria Antonietta Gambacorta, Luca Indovina, Lorenzo Placidi

**Affiliations:** ^1^Fondazione Policlinico Universitario ‘‘A. Gemelli’’ IRCCS, Rome, Italy; ^2^Radiotherapy Department, Università Cattolica del Sacro Cuore, Rome, Italy

**Keywords:** gas pockets, relative electron density, MRgRT, online adaptive radiotherapy, dosimetry

## Abstract

**Introduction:**

Contouring of gas pockets is a time consuming step in the workflow of adaptive radiotherapy. We would like to better understand which gas pockets electronic densitiy should be used and the dosimetric impact on adaptive MRgRT treatment.

**Materials and methods:**

21 CT scans of patients undergoing SBRT were retrospectively evaluated. Anatomical structures were contoured: Gross Tumour Volume (GTV), stomach (ST), small bowel (SB), large bowel (LB), gas pockets (GAS) and gas in each organ respectively STG, SBG, LBG. Average HU in GAS was converted in RED, the obtained value has been named as Gastrointestinal Gas RED (GIGED). Differences of average HU in GAS, STG, SBG and LBG were computed. Three treatment plans were calculated editing the GAS volume RED that was overwritten with: air RED (0.0012), water RED (1.000), GIGED, generating respectively APLAN, WPLAN and the GPLAN. 2-D dose distributions were analyzed by gamma analysis. Parameter called active gas volume (AGV) was calculated as the intersection of GAS with the isodose of 5% of prescription dose.

**Results:**

Average HU value contained in GAS results to be equal to -620. No significative difference was noted between the average HU of gas in different organ at risk. Value of Gamma Passing Rate (GPR) anticorrelates with the AGV for each plan comparison and the threshold value for GPR to fall below 90% is 41, 60 and 139 cc for WPLANvsAPLAN, GPLANvsAPLAN and WPLANvsGPLAN respectively.

**Discussions:**

GIGED is the right RED for Gastrointestinal Gas. Novel AGV is a useful parameter to evaluate the effect of gas pocket on dose distribution.

## 1 Introduction

Online adaptive MRI-guided radiotherapy (MRgRT) has many advantages for the patient (1–5), one of the most important is the ability to provide a treatment plan that is best tailored to the daily anatomical situation of the patient. Online adaptive treatments involve a multidisciplinary approach in the MR-Linac treatment control room: Radiation Therapists (RTT) for positioning, Radiation Oncologists (RO) for recontouring and Medical Physicists (MP) for evaluating the eventual changes in electron density (ED) map that could affect plan recalculation, before re-optimize (if needed) the dose distribution. One of the tasks of MP is to verify the accuracy of the. This map is generated from the simulation CT and is used as a “ED-model” for the patient in the dose calculation process. The simulation CT, being performed a few days before the treatment, has a sensitivity to the day-to-day situation that can vary, as the patient may go through anatomical variations. Indeed, among the inter- and intra-fraction anatomical variations that may occur, the one that has the greatest effect on the electron density map, is the formation and displacement of abdominal and pelvic gas pockets. These can alter the patient’s anatomy by displacing both targets and organs at risk (OAR) in their proximity (6), but most importantly creating interfaces with very different electron densities, affecting dose distribution (7–9). During the online adaptive workflow, MP is concerned, in the preliminary phase, with the recontouring volumes useful for dose optimization including gas pockets. In the current adaptive workflow, the simulation CT can be rigidly or deformably registered with the daily MRI scan to obtain the updated electron density map. In the rigid workflow it is obvious that the re-countouring of the gas pockets must be done manually, but this is also the case in the deformable workflow if the gas pockets deviate greatly in volume and position since the deformable image registration (DIR) algorithm cannot reproduce them correctly. Accurate and complete contouring of the gas pockets position and size plays an important role in the process of adaptive radiotherapy especially since its purpose is to provide a treatment plan that included the daily variation in electron densities due to daily anatomy changing. Contouring is still a time-consuming process, even with the aid of modern automatic contouring tools. Therefore, it is important to understand when contouring is necessary and when it can be overlooked. The present literature mainly focusing on the issue of the electron return effect (ERE) with phantoms studies, CTs with *ad-hoc* synthetic gas pockets and Monte Carlo simulations (7, 9–13). In the few clinical published papers, the authors describe the effect of gas pocket only varying the metrics of the dosimetric analysis in terms of gamma passing rate (GPR), or by conformation indices, or comparing dose volume histograms (DVHs) of targets and OARs, reporting also very different results (8, 14–16). With this study, we would like to gain a better understanding of the nature of electronic densities of gas pockets and their dosimetric impact on a MRgRT treatment in a hybrid 0.35 T MRI-Linac. The aims of this study are:

- to evaluate a CT-derived gas pockets RED, considering that in the clinical practice air RED is used for gas pockets RED override
- to evaluate the differences in gas pockets RED with the respect to the specific OARs in which they are contained
- to quantify the dosimetric impact (both on targets and OARs) of gastrointestinal (GI) gas pockets RED override with different REDs
- to define a quantitative parameter able to describe such dosimetric impact

## 2 Materials and methods

### 2.1 Evaluation of gas pockets relative electron density map

**Figure 1:**
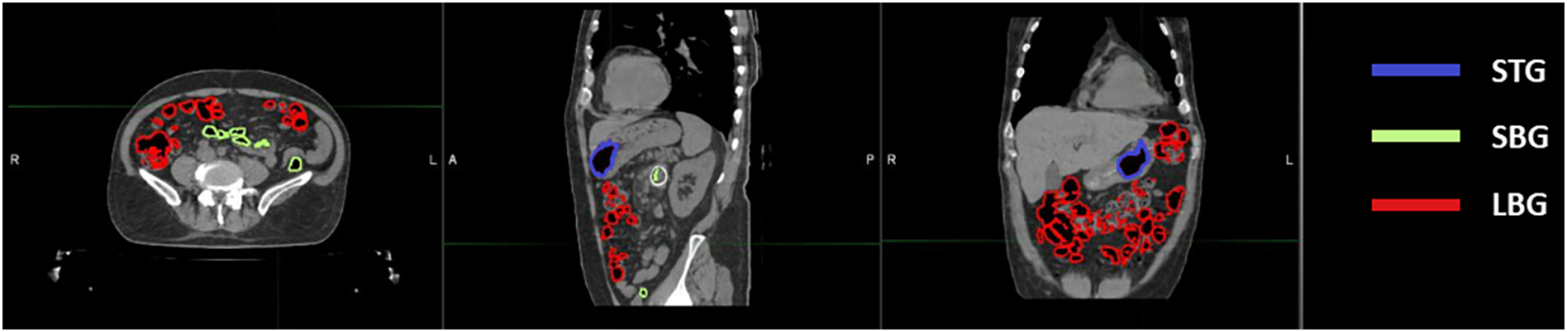
Location of abdominal gas volumes divided by the organ at risk in which they are located. A patient’s CT is shown with gas pockets in the stomach highlighted in blue, those in the small bowel in green and those in the large bowel in red.

Twenty-one CT scans, one for each different patient undergoing SBRT for gastrointestinal lesion were retrospectively evaluated. All the scans were acquired using the radiotherapy department simulation CT scanner (Discovery Optima, GEhealthcare, Madison, WI) with the same standard GI protocol. Our center’s protocol provides a CT acquisition with 120 kV, 135 mA, slice thickness 1.25 mm, in plane resolution 1.27 x 1.27 mm^2^ and the maximum field of view for all GI patients. The following anatomical structures were contoured during the treatment planning process: Gross Tumour Volume (GTV) and relative 3 mm isotropic expansion (PTV), the Gastrointestinal Organs at Risk (GIOARs), including stomach (ST), small bowel (SB), large bowel (LB) and gas pockets in the abdominal cavity (GAS). Three additional specific volumes of gas pockets were also defined and contoured: the intersection of GAS volume with each GIOAR obtaining stomach gas volume (STG), small bowel gas volume (SBG) and large bowel gas volume (LBG) (**Figure 1**). We did not deem it appropriate to add the duodenum to the list of GIOARs containing gas pockets due to the scarcity of gases present within its lumen in the sample of patients analyzed. All contouring were performed by a radiation oncologist with at least 5 years of experience. The normality of the distribution of the mean values of the Hounsfield Units (HU) of pixels contained in GAS, STG, SBG and LBG volumes for all CT scans was tested singularly using a Kolmogorov-Smirnov test (p=0.05). Mean value of HU values of the pixels contained in GAS structure of all patients was converted in RED using the calibration curve of the CT scanner and the obtained value has been named as Gastrointestinal Gas relative Electron Density (GIGED). Finally, differences of mean values of HU of the pixels included in GAS, STG, SBG and LBG using a Wilcoxon-Mann-Whitney test for unpaired samples with a threshold p-value of 0.05 were computed.

### 2.2 Evaluation of dosimetric impact

**Figure 2:**
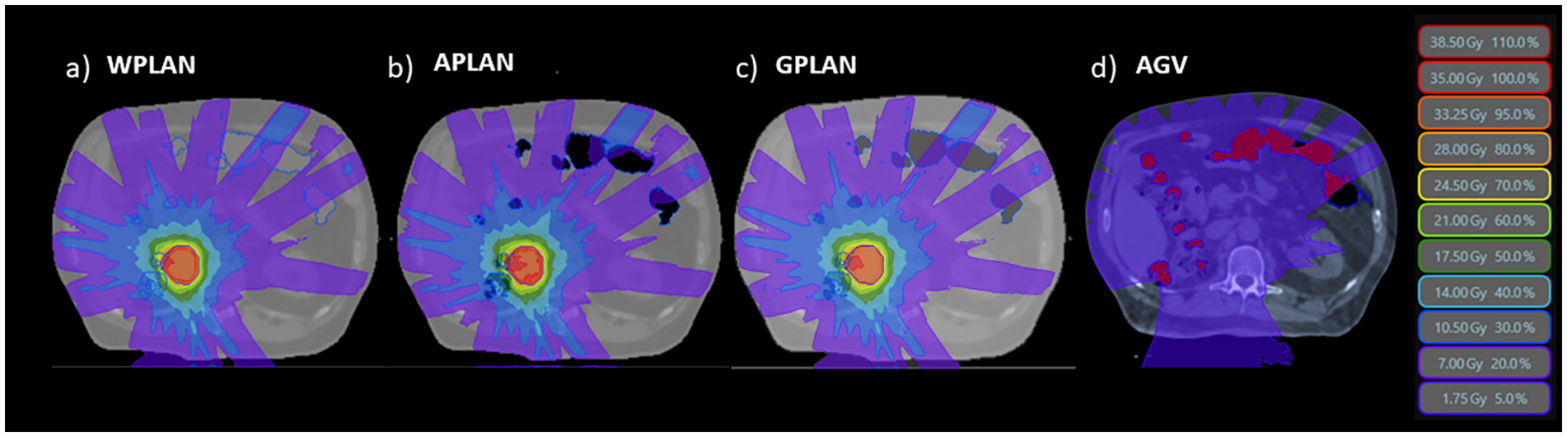
The three different dose distributions due to the recalculation of the treatment plan on the different electronic density maps generated by overwriting the volume of gas pockets with RED of water (**A** - WPLAN), RED of air (**B** - APLAN) and GIGED (**C** - GPLAN). In **D** we show the 5% prescription dose isodose intersecting the AIR structure defining the AGV parameter.

For each patient, three different treatment plans were calculated starting from the same clinical plan. The same fluences of the clinical plan were re-computed without any plan optimization, only editing the GAS volume RED that was overwritten with:

1 - the conventional AIR relative electron density value (0.0012, standard TPS value) (APLAN)
2 - the water electron density value (1.000) (WPLAN)
3 - the GIGED value (GPLAN)

generating respectively the APLAN, WPLAN and the GPLAN. WPLAN aims to simulate the extreme-case scenario of the patient presenting completely without air pockets. All clinical initial plans were calculated using the MRIdian (ViewRay Technologies Inc., Oakwood Village, OH) TPS by an experienced medical physicist. The planning was realized using the MRIdian Planning Technique (MPT) (17, 18) a particular technique for robust online adaptive planning. The plans were optimized using a standard template for the positioning and number of the Linac gantry angles. Twenty-four beams were equally distributed around the patient, except for two small sectors of about 20 degrees positioned at about 120 and 240 degrees where no radiation beams were present to avoid couch edges. All treatment plans dose prescriptions were at the 80% isodose and ranged from 35 to 40 Gy. Dose optimization for IMRT step-and-shoot treatments was then performed using the Kawrakow Monte Carlo (KMC) algorithm (19) on the MRIdian TPS (2500000 histories, dose grid 1.0 x 1.0 mm^2^, 1% of recalculation uncertainty (20)). 2-D dose distributions obtained for APLAN, WPLAN and GPLAN were compared to each other in terms of gamma analysis (1%/1mm, threshold 10%) (21, 22).. The thresholds of the gamma analysis were chosen to be as tight as possible, being the calculation grid of both dose distributions of 1 mm and the recalculation error of the TPS being estimated at 1%. 2-D gamma analysis was performed separately for the three projections of dose on the three orthogonal planes passing through the centroid of the GTV. Mean value of the three relative gamma passing rates was then considered to evaluate differences in dose distribution. In addition, a parameter called active gas volume (AGV) was introduced and calculated as the intersection of the GAS structure with the isodose of 5% of prescription dose (**Figure 2**). AGV aims to evaluate and quantify the volume of gas invested by beam path. The Pearson correlation index was computed to verify the correlation between GPR values and the AGV parameter. Finally, DVH analysis was performed extracting the following values to assess dose distribution variation: the percentage volume of PTV covered by 95% of the prescription dose (PTV_V95), minimum dose to PTV as the isodose level that covers the 98% of the PTV volume (PTV_D98), the mean dose to PTV (PTV_D50) and the maximum dose to PTV as the isodose level that covers the 2% of the PTV volume (PTV_D2). Minimum, mean and maximum dose values were then extracted from DVH for the three GIOARs as done for the PTV. The differences of these extracted values for each plan comparison were computed and analyzed.

### 2.3 Dependence of electron return effect on relative electron density

**Figure 3:**
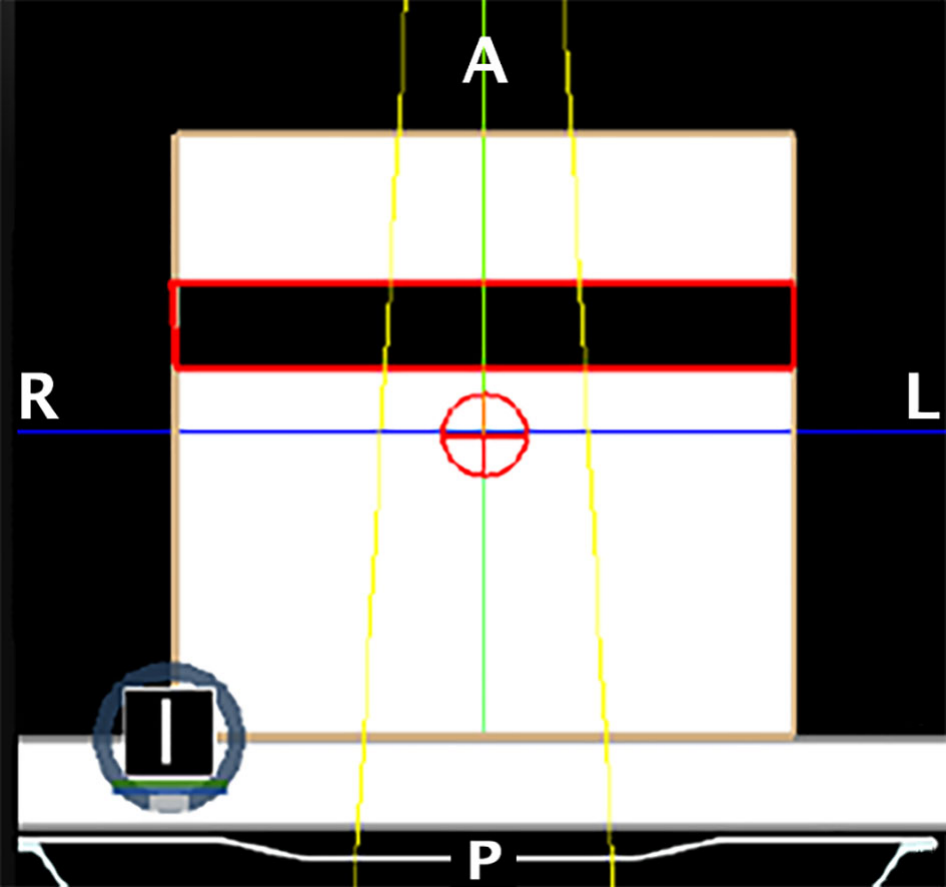
Setup of the phantom for the ERE dependency on the interface’s RED experiment. The crosshair sign in red represents the isocenter of the single fixed conformal beam (in yellow). The dark zone contoured in red represents the gap with variable RED.

In order to evaluate the dependence of the ERE on the electron density of the interfaces, we conducted a further in silico study. This evaluation better explain and support the obtained results. A Monte Carlo calculation was carried out using the MRIdian TPS (ViewRay Technologies Inc., Oakwood Village, OH) to evaluate the effect of a fixed conformal beam with a 10x10 cm^2^ field size delivering 10 Gy at the isocentre onto a synthetic cubic phantom. The phantom consisted of a 30x30x30 cm^3^ solid water block that has a medium interface inside which its relative electron density can be varied (**Figure 3**). The density-varying gap zone is located 7.5 cm from the top surface of the cubic phantom while the isocentre is 15 cm from the top surface. Eleven different dose distributions were calculated, with a dose grid resolution of 1.0x1.0 mm^2^, by varying the relative electron density of the gap from air (0.0012) to water (1.0) in steps of 0.1. The axial projections of these dose distributions were compared to that obtained using the RED of air in terms of 2-D gamma analysis with 1%/1mm threshold. We also extracted the percentage dose depth (PDD) on the beam central axis for each calculated dose distribution in order to visualize the differences in terms of ERE.

## 3 Results

### 3.1 Evaluation of gas pockets relative electron density

**Figure 4:**
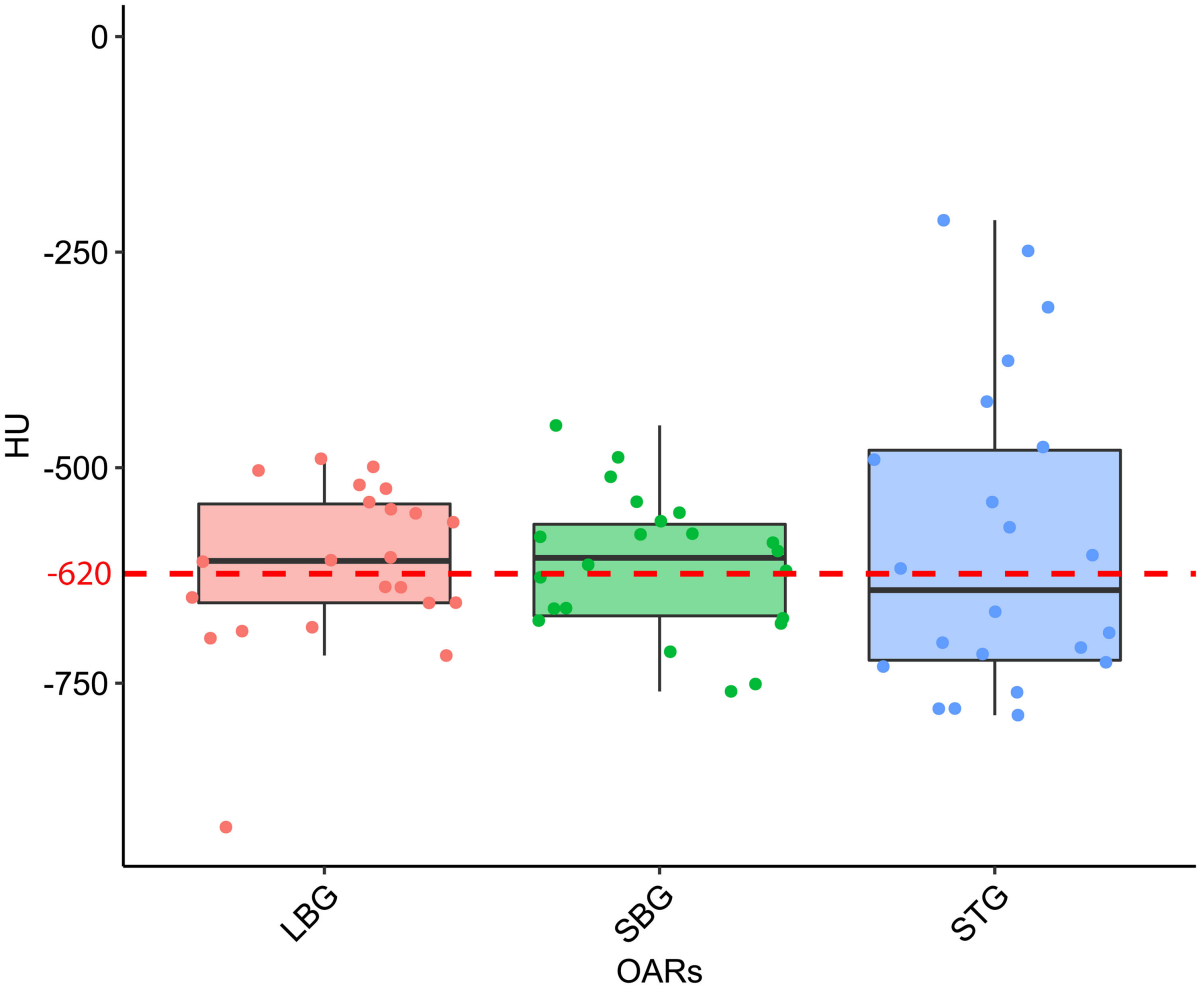
Boxplot distribution of the mean Hounsfield Units (HU) values distribution of the Large Bowel Gas (LBG), Small Bowel Gas (SBG) and Stomach Gas (STG) structures with their relative Wilcoxon test p-values. The dashed red line represents the mean HU value of the entire GAS structure, namely -620 HU.

The distribution of the mean values of the HU of pixels contained in both GAS, STG, SBG and LBG structures for all CT scans results to be normally distributed (p-values are 0.71, 0.46, 0.97, 0.94 respectively). Considering all 21 CT scans the mean HU value contained in of GAS structure results to be equal to -620 HU with a standard deviation (SD) of 90 HU. Regarding the different organs we have obtained ( ± SD in parenthesis) -610 ( ± 100) HU for LBG, -590 ( ± 180) HU for STG and -610 ( ± 80) HU for SBG. P-values of statistical test calculated for STG vs. LBG comparison is 0.62 while STG vs. SBG and SBG vs. LBG comparisons are 0.60 and 0.74 respectively (**Figure 4**).

**Figure 5:**
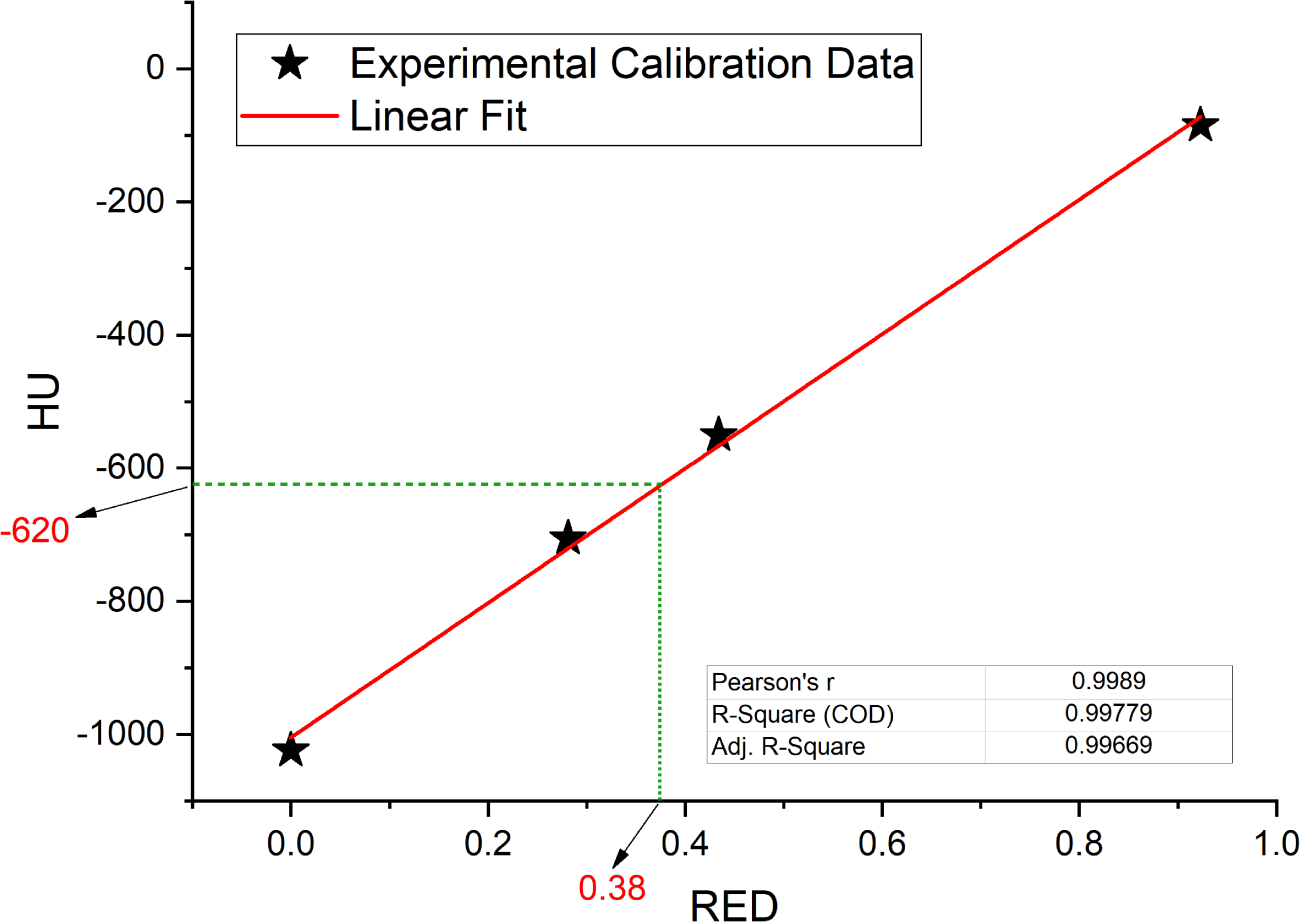
Interpolated value for relative electron densities (RED) from the mean value of Hounsfield Units (HU) contained in GAS volume, interpolation on linear fit (red line) of experimental data collected in the simulation CT calibration curve.

Linear fit of experimental data acquired in the calibration of the simulation CT scanner are reported in **Figure 5** together with fit parameters. Only the part of the calibration curve between 0 and -1000 HU has been considered for this study. Linear fit was used to calculate the GIGED value that result to be equal to 0.38 for a HU value of -620.

### 3.2 Evaluation of dosimetric impact

**Figure 6:**
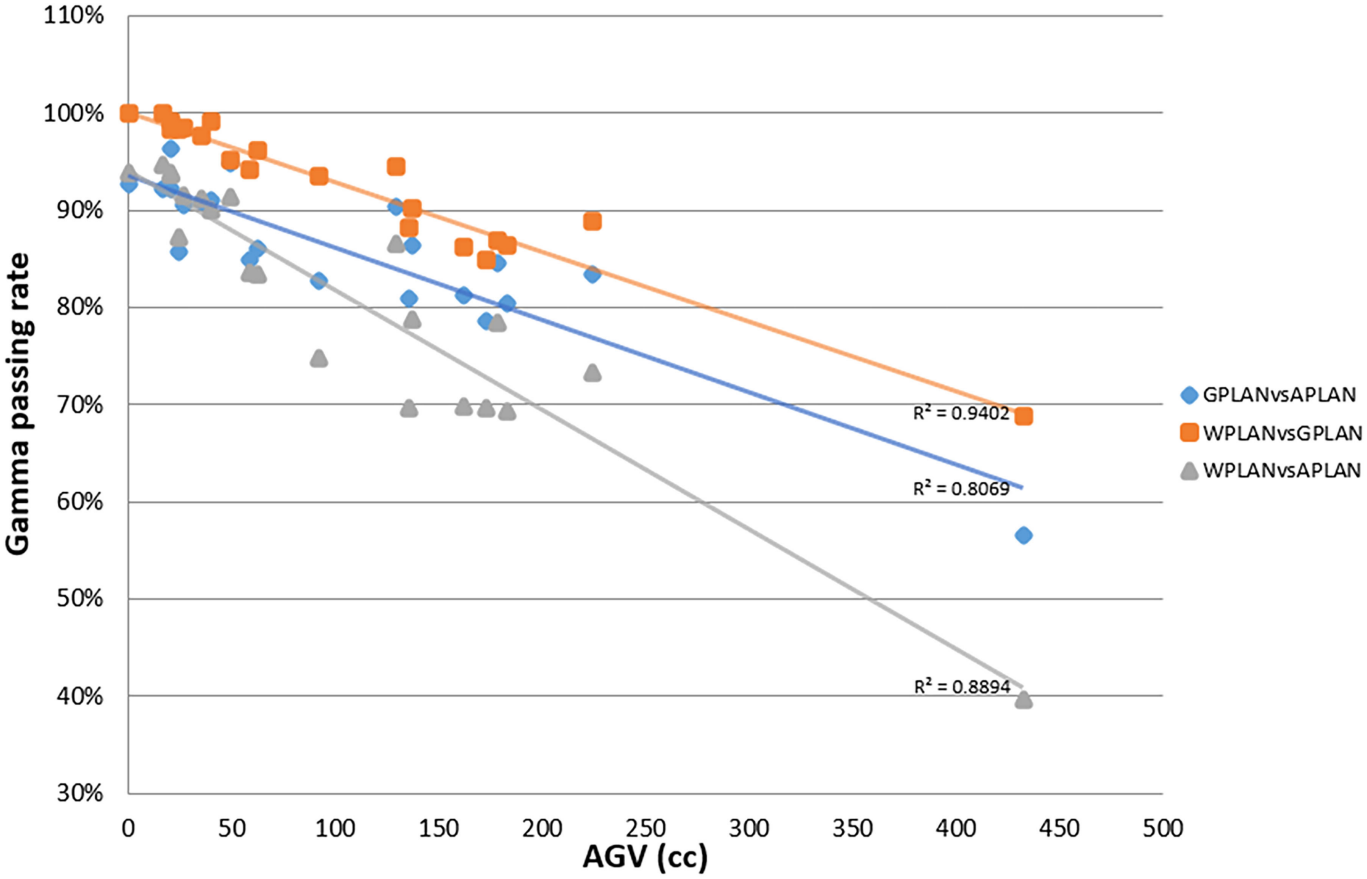
Plot of gamma passing rate (GPR) trend as a function of active gas volume (AGV) parameter (in cc) for comparisons between plan calculated overwriting gas pockets electron density with water’s one (WPLAN), air’s one (APLAN) and the one calculated in this study (GPLAN). The solid line represents the linear fit and the relative R2 values are shown (GPLAN vs. APLAN in blue, WPLAN vs. GPLAN in orange and WPLAN vs. APLAN in grey).

**Figure 7:**
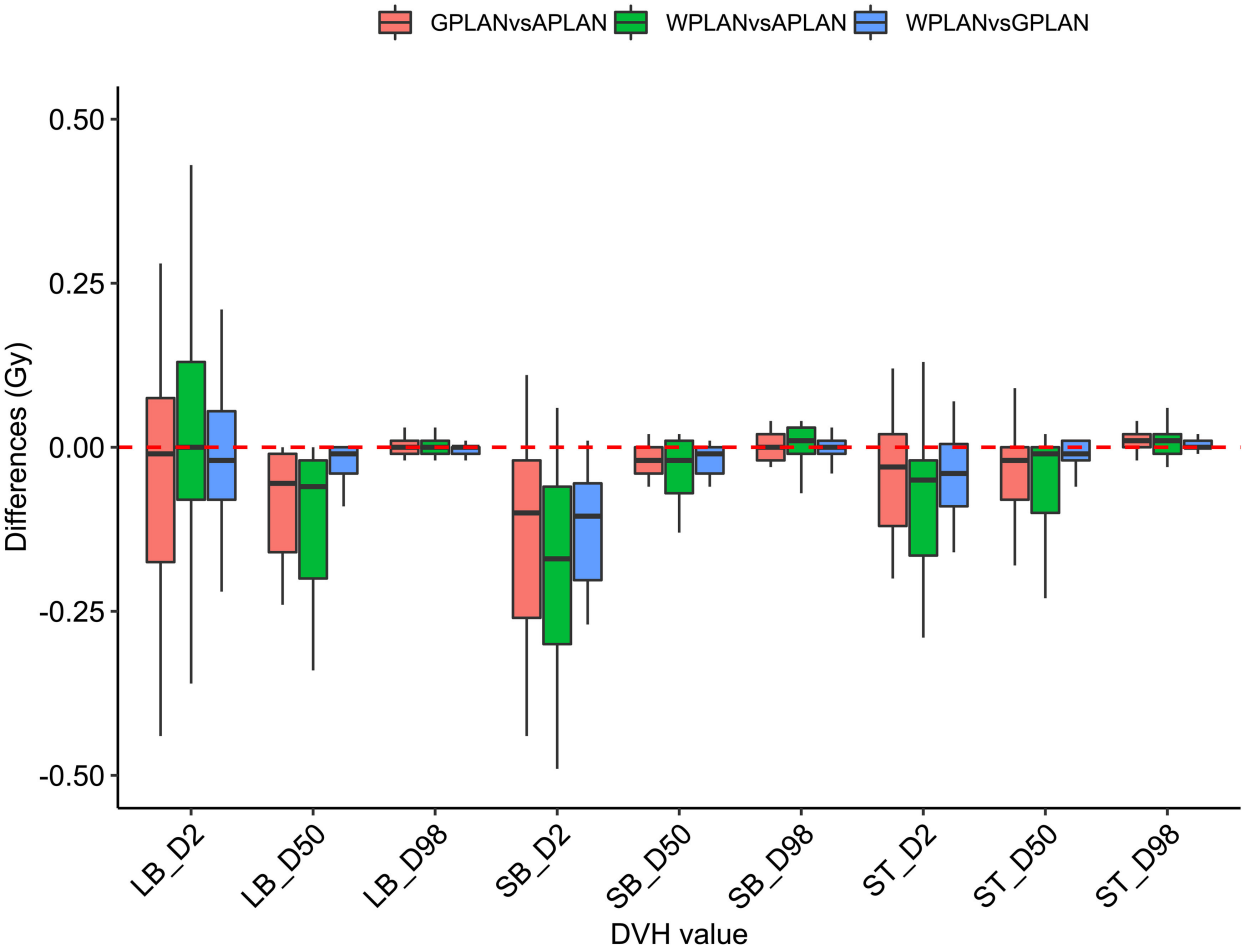
Boxplots of the distributions of the differences (in Gy) in the dose volume histogram (DVH) extracted values for D2, D50 and D98 of different organs at risk (large bowel (LB), small bowel (SB) and stomach (ST)) for different plan comparisons (in red GPLAN vs. APLAN, in green WPLAN vs. APLAN and in blue WPLAN vs. GPLAN).

**Figure 8:**
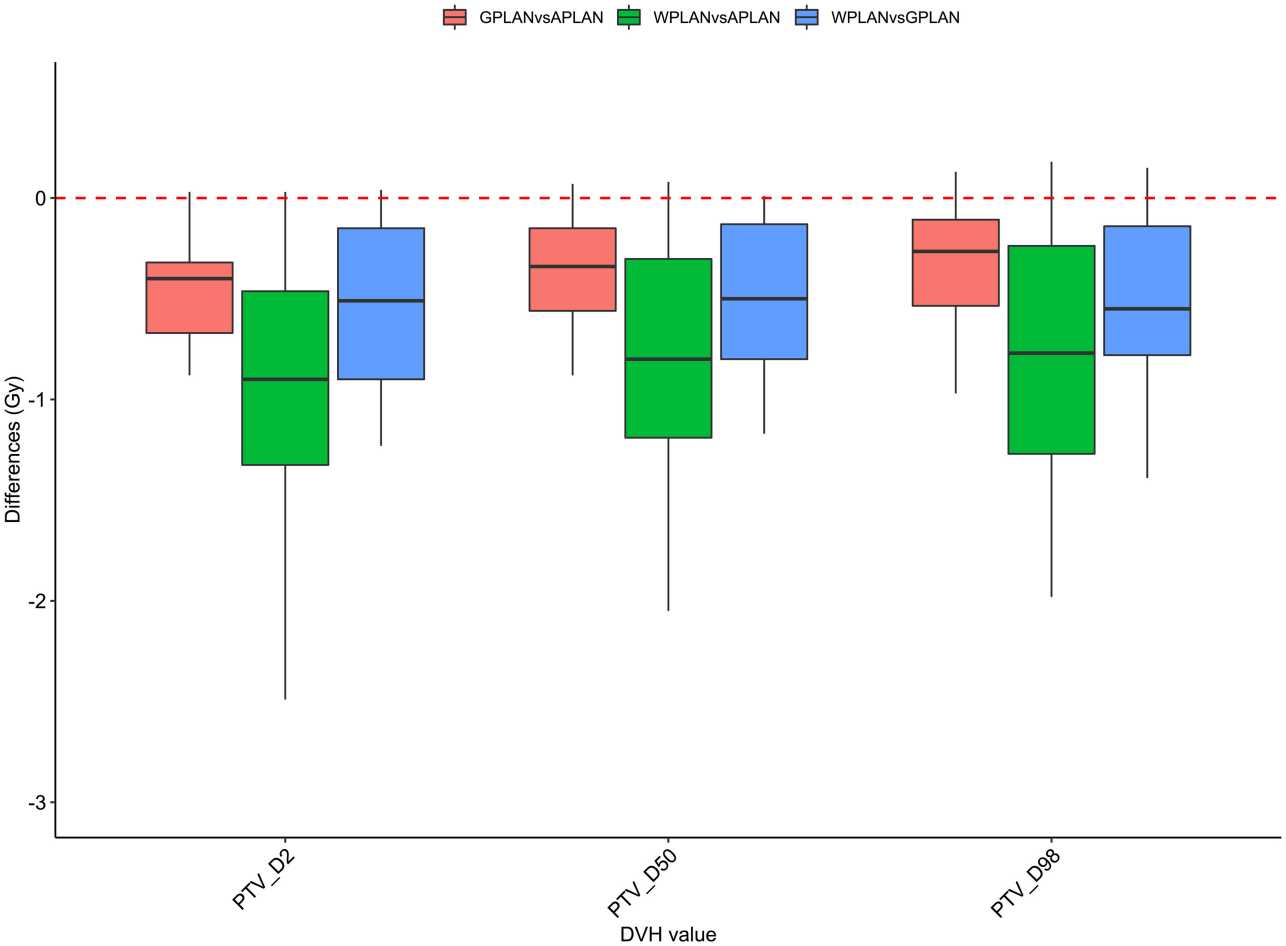
Boxplots of the distributions of the differences (in Gy) in the dose volume histogram (DVH) extracted values for D2, D50 and D98 of PTV for the different plan comparisons (in red GPLAN vs. APLAN, in green WPLAN vs. APLAN and in blue WPLAN vs. GPLAN).

**Figure 9:**
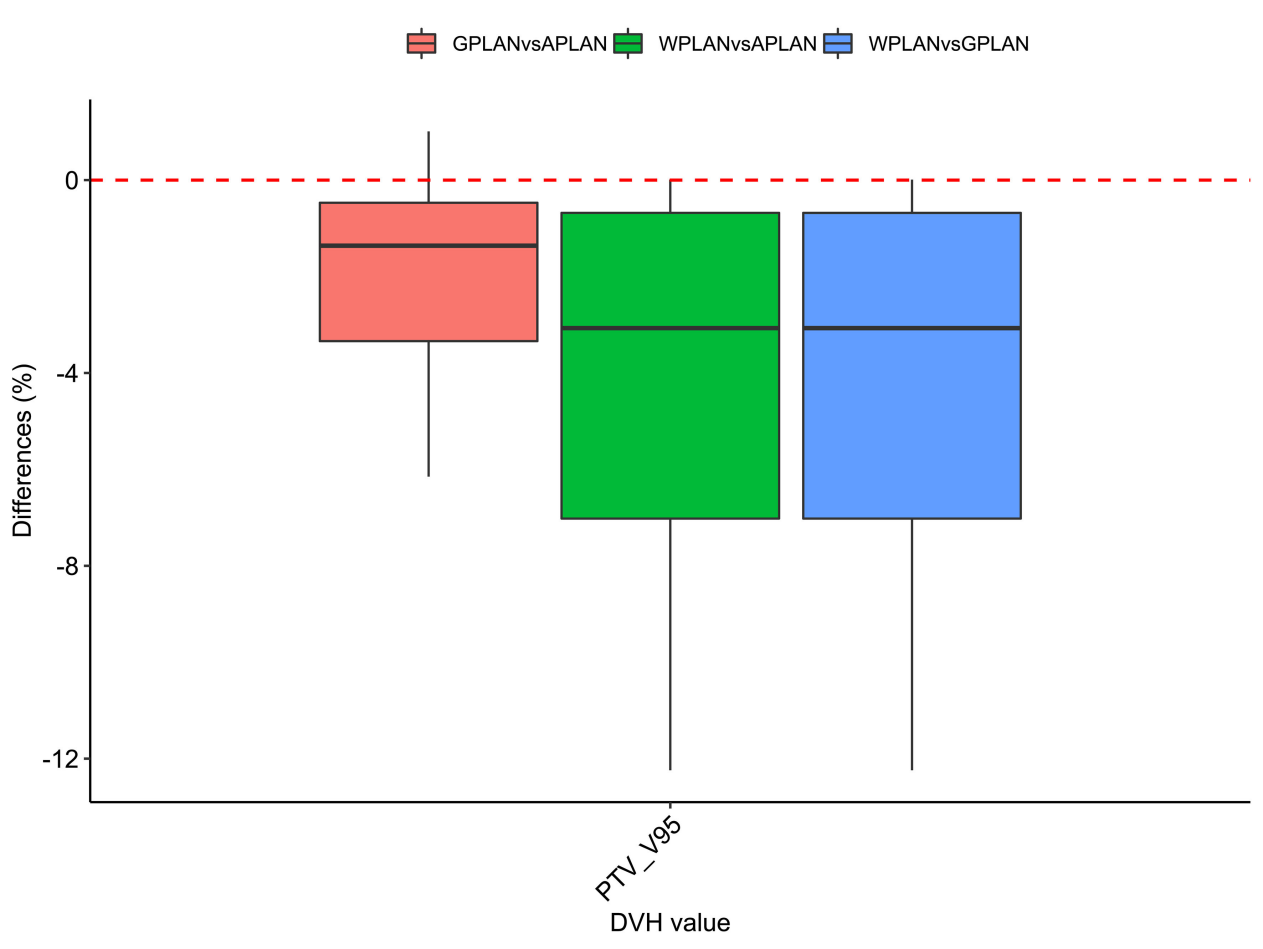
Boxplots of the distributions of the percentage differences in the dose volume histogram (DVH) extracted values for V95 of PTV for the different plan comparisons (in red GPLAN vs. APLAN, in green WPLAN vs. APLAN and in blue WPLAN vs. GPLAN).

Results for mean values of gamma passing rates (GPR) are summarized in **Table 1** as well as the calculated values for AGV in cc. For the comparison of WPLAN and APLAN values of GPR range from 40% to 95% with a mean value of 81% while for WPLAN vs GPLAN values range from 69% to 100% with a mean value of 93%. For what concerns GPLAN vs APLAN we have a minimum value of GPR of 57% and a maximum of 96% for a mean value of 86%. Values are plotted in **Figure 6** once sorted by increasing AGV value. In addition, we have added lines on the graph for a linear fit of the data for each comparison with the relative R squared value. Based on these linear fits, it’s possible to interpolate, for each comparison, the threshold value of AGV for which the GPR falls below 90%, which is the GPR threshold value recommended by AAPM TG 218 for gamma tests for IMRT treatment plans with thresholds of 3% in dose difference and 2 mm distance to agreement (21). Those values result to be at least 41, 60 and 139 cc for WPLAN vs APLAN, GPLAN vs APLAN and WPLAN vs GPLAN respectively. Pearson’s correlation coefficient between GPR and AGV results to be -0.97, -0.94 and -0.89 for WPLAN vs. GPLAN, WPLAN vs. APLAN and GPLAN vs. APLAN respectively, demonstrating a significant inverse correlation between the two variables. Differences in values extracted from DVH for each comparison of plans are summarized in **Figures 7**–**9** (boxplot are shown without outliers). **Figure 7** shows the DVH differences (D98, D50 and D2) in Gy for the three GIOARs evaluated that remain between -1.20 and 1.95 Gy. In **Figure 8** are described the PTV’s maximum, minimum and mean dose difference. In this case we find that difference values range from 0.50 to -4.13 Gy. **Figure 9** shows difference in PTV coverage: values range between 1.01% to -21.75%. With regard to the patients analyzed, the GTV volume has an average value of 26.28 cc with a maximum of 74.7 cc and a minimum of 1.1 cc, the standard deviation is 18.66 cc. For the PTV we have an average of 42.67 cc with a maximum of 110.1 cc and a minimum of 3.0 cc, the standard deviation is 26.66 cc.

**Table 1:** Values of the average gamma passing rate (GPR) (1%, 1mm) for comparisons between plan calculated overwriting gas pockets electron density with water’s one (WPLAN), air’s one (APLAN) and the one calculated in this study (GPLAN) for all patients analyzed.

Mean GPR (1%,1mm)				
Patient ID	WPLAN vs APLAN	WPLAN vs GPLAN	GPLAN vs APLAN	AGV (cc)
Patient 1	73%	89%	83%	224.58
Patient 2	94%	100%	93%	0.36
Patient 3	94%	99%	92%	20.43
Patient 4	83%	96%	86%	62.47
Patient 5	78%	87%	85%	178.49
Patient 6	84%	94%	85%	58.44
Patient 7	90%	99%	91%	40.04
Patient 8	70%	88%	81%	135.64
Patient 9	79%	90%	86%	137.2
Patient 10	87%	98%	86%	24.26
Patient 11	87%	94%	90%	129.71
Patient 12	91%	98%	91%	35.44
Patient 13	95%	100%	92%	16.46
Patient 14	94%	98%	96%	20.94
Patient 15	70%	86%	81%	162.14
Patient 16	69%	86%	80%	183.48
Patient 17	75%	94%	83%	91.87
Patient 18	40%	69%	57%	432.63
Patient 19	92%	99%	91%	26.72
Patient 20	91%	95%	95%	49.09
Patient 21	70%	85%	79%	172.98

Mean GPR is the result of averaging the individual 2-D GPRs calculated in the three orthogonal planes passing through the centroid of the GTV. In the last column the value of the active gas volume (AGV) (in cc) parameter calculated as the intersection of the GAS structure with the 5% prescription dose isodose.

### 3.3 Dependence of electron return effect on relative electron density

**Figure 10:**
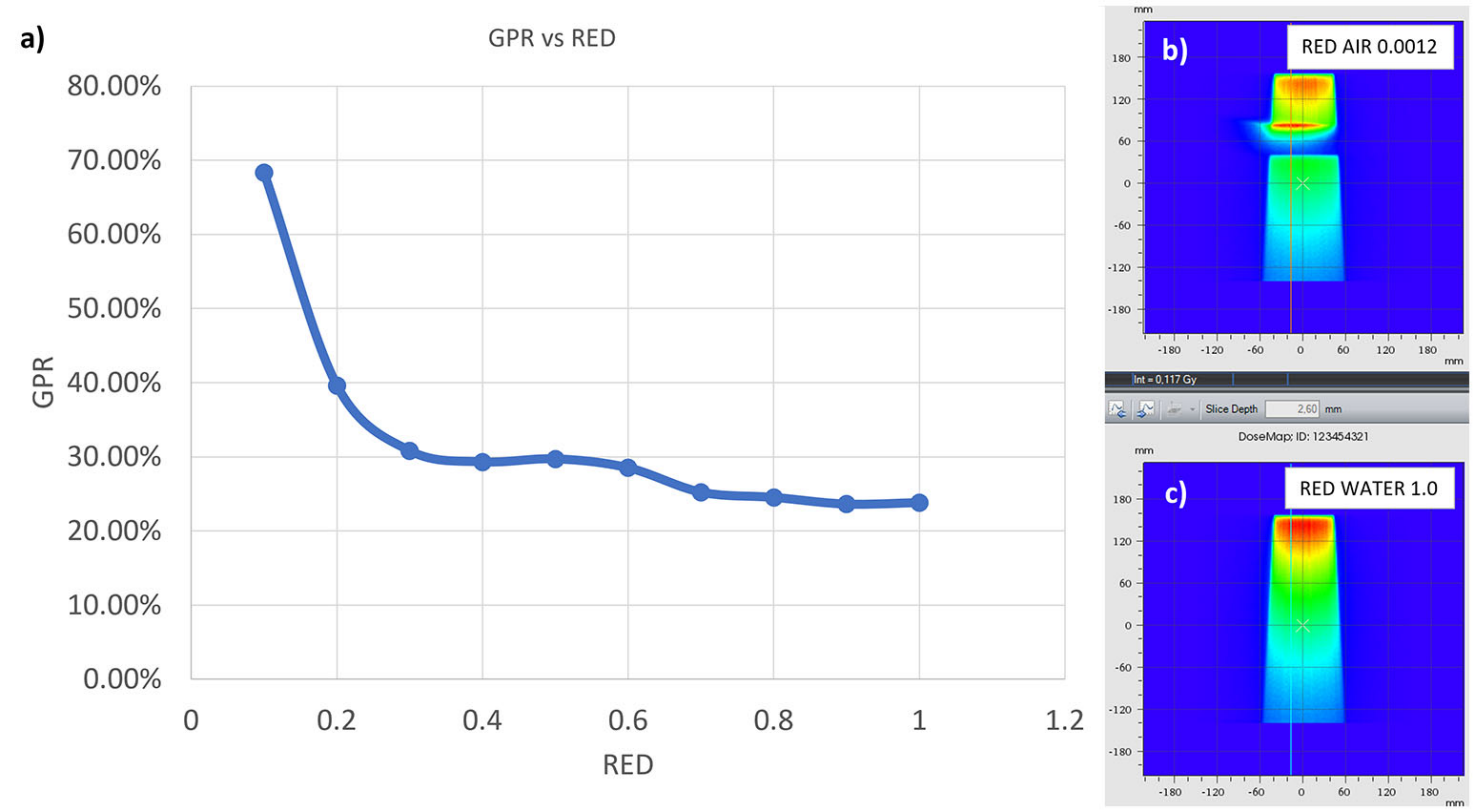
GPR trend as a function of the RED of the interface in the synthetic phantom **(A)**. In **(B, C)** the axial projection of the dose distribution in the phantom generated by a 10x10 cm^2^ field in the presence of an air gap **(B)** and with the gap filled with water **(C)**.

**Figure 11:**
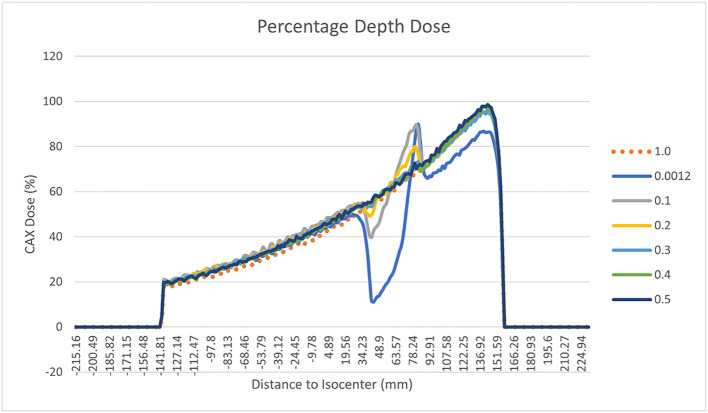
Percentage Depth Dose trend measured on the central field axis and normalised to the maximum dose for different RED of the gap of the synthetic phantom. The various curves with different colours relate to the different RED values of the gap. In particular the orange dotted line is related to water’s RED value.

The results of the gamma analysis performed are reported in **Table 2**. In the left column are reported the values of the density gap’s RED in the phantom used for the study, while in the right column, the corresponding values of GPR for the comparison with the dose distribution calculated for the air’s RED. The values change continuously from 68.3% for a RED of 0.1 to a value of 23.8% for a RED of 1.0. The trend of GPR values as a function of RED is plotted in panel “a” of **Figure 10**: the greatest variation in GPR occurs between the values of 0.1 and 0.3 of RED, with a difference of approximately 37%, while at higher RED values, GPR remains nearly constant. In panels “b” and “c” of **Figure 10** are reported the axial projections of the dose distribution calculated for REDs of 0.1 and 1.0, respectively, showing how ERE changes the dose distribution. **Figure 11** shows the central axis dose rate (CAX), normalised to the global maximum value, as a function of the distance from the isocentre of the radiation beam. The various curves with different colours relate to the different RED values of the gap. For RED values between 0.0012 and 0.4 the distortions due to ERE are less evident as the RED increases, while for the value of 0.5 (dark blue solid line) the graph already overlaps almost exactly with the curve relative to the RED value of water (dashed orange line). Higher RED values are not plotted in the graph for clarity.

**Table 2:** Values of the GPR of the 2-D gamma analysis for comparison between variable RED dose distribution and Air RED dose distribution.

RED	GPR
0.1	68.30%
0.2	39.60%
0.3	30.80%
0.4	29.30%
0.5	29.70%
0.6	28.50%
0.7	25.20%
0.8	24.50%
0.9	23.60%
1	23.80%

Dose distribution have been calculated by Monte Carlo using a synthetic phantom (**Figure 3**).

## 4 Discussion

### 4.1 Gas pockets RED

Considering the results obtained in the session on the evaluation of gas pockets RED, we demonstrated that the gas contained in the examined OARs does not show statistically significant difference in terms of RED. It therefore turns out to be a legitimate choice to treat the GAS volume as a single volume and to define its RED by taking the average value of the HUs relative to the pixels contained in the GAS volume. The result shows that the RED of the intestinal GAS pockets should not set equal to air’s RED but a higher RED (0.38). All the previously published studies suggested to overwrite the electronic densities of the daily recontoured gas pockets with RED of air. Such approach increase the uncertainty in the daily adapted dose recalculation since the RED of air is an order of magnitude lower than that of the gas pockets (8, 14, 15). In **Figure 6**, the fits have no pretensions to assert that the model describing the trend of GPR vs. AGV is linear, although the trend has an acceptable R^2^ in the case of WPLAN vs. GPLAN. Nevertheless, it graphically visualizes the decreasing trend of the GPR function when AGV increases. In the comparison between the recalculated plan with water RED (WPLAN) and the one overwriting the gas pockets RED with GIGED (GPLAN), it is found that the gamma passing rate drops below 90% when the AGV is greater than 139 cc. Therefore, considering the results of this study, it is our opinion that overwriting the RED of gas pockets with air RED can lead to a dosimetric error and should be better to overwrite it with GIGED. The greater the AGV, the greater the dosimetric error will be.

### 4.2 Dosimetric analysis

Considering the three trend lines shown in **Figure 6**, the one with the steepest slope is the one comparing WPLAN and APLAN due to the relevant difference in the REDs used. When compared GPLAN and APLAN, and WPLAN and GPLAN, the slopes are less steep: indeed GIGED is about in the middle between the REDs of air and water. Considering the results obtained in this work in our study concerning the dependence of ERE on the RED of the gap, it can also be understood how, depending primarily on ERE, the difference between GPLAN and APLAN is greater than the difference between GPLAN and WPLAN even though the difference between GIGED and air RED is less than between GIGED and water RED. These differences are also reinforced in **Figures 7**–**9** where the WPLAN vs. APLAN comparison (in green) always shows the highest absolute values. **Figure 7** depicts and highlights that the largest differences are found in the high and medium dose regions. For low dose region a minor variance is visible and the results show no differences. Differences of the maximum, mean, and minimum dose have a negative trend in the PTV, as well as the target coverage which decreases as the RED of the GAS volume decreases. At present, we cannot justify why, quite counterintuitively, it would appear that the difference in the very high dose zone (PTV) is smaller between APLAN and GPLAN than between GPLAN and WPLAN, although it must be said that these differences are not statistically significant. Further investigation is needed to unravel this interesting topic.

### 4.3 Comparison with past studies and future implications

Estabrook et al. (16) recalculated the dose distribution using a daily CBCTs, founding an average reduction in target coverage of 3.3%. This target coverage reduction is comparable to the one found in this work, although they found no correlation between the dose covering the 100% of the PTV and the volume of gas pockets. Jin et al. (14) proposed a retrospective MRgRT analysis on 5 cases of pancreatic cancer patients in which the treatment plans of each fraction are recalculated using gas volume contours on the MRs and overwritten with air density. In this paper, the results obtained describe the variation of dosimetric parameters based on intra-fraction variations in the volume and position of the gas pockets. As far as the PTV is concerned, no significant dosimetric changes were found, with a maximum change of 1 Gy in absolute dose. Jin et al. do not present any data or analysis to evaluate the dosimetric impact as a function of the volume variations of the gas pockets. The work carried out by Su et al. within the framework of the SMART protocol (23), reports the results obtained in a dosimetric study where air pockets were recontoured during adaptive MRgRT of 30 patients undergoing SBRT on pancreas in 5 fractions. Homogeneity index (HI), Conformity Index (CI) and Conformity Number (15) are compared for the resulting plans that benefited from the recontouring of gas pockets with the corresponding correction of the electron densities and those that were calculated leaving the CT uncorrected. The reported differences, in terms of D98, D95, D50 and D2 for both PTV and GTV, are not significant. Also in the study by Su et al., no correlations between gas pockets and dosimetric variation has been found. This is due to the small gas volume variation (order of a few cc) that do not allow a direct comparison with our study. This study has some limitations that have to be considered. The number of patients enrolled in this study could be increased even if it a well representative dataset for such analysis, also in comparison within the published literature. Afterwards, the results obtained in this study are related to the calibration curve of the simulation CT of our center: it could be interesting to extend the analysis to other centers. Obviously, with the advent of synthetic CT (24, 25), the considerations and results obtained may be overcome as this technology will allow an automatic, fast and accurate electronic densities correction based on to the daily anatomy although no algorithms sophisticated enough to generate synthetic CTs from MR scans that accurately reproduce the position and volume of both abdominal and pelvic gas pockets can be found in the state of the art. In conclusion this study, as far as our knowledge, is the first that provide a quantitative parameter for correlation between gas pocket s’ volume and its dosimetric effect. No difference in terms of HU for the gas contained in the various GI organs was found and the average HU value is -620 which corresponds to a RED of 0.38 (GIGED), according to our center’s simulation CT calibration curve. The dosimetric results showed that correlations are found between the reduction of the gamma passing rate and the value of the measured AGV. Therefore, AGV is a useful parameter to evaluate the effect of gas pocket on the clinical dose distribution in MRgRT GI treatments. In particular, we found that, using the gamma passing rate metric, a difference of 139 cc of AGV is sufficient to create a significant difference in dose. This value could be used as a threshold for deciding whether or not to recontour gas volumes in the abdomen during an online adaptive workflow.

## Data availability statement

The raw data supporting the conclusions of this article will be made available by the authors, without undue reservation.

## Ethics statement

Ethical approval was not required for the study involving humans in accordance with the local legislation and institutional requirements. Informed consent was obtained from all individual participants included in the study.

## Author contributions

MN: Conceptualization, Data curation, Investigation, Methodology, Writing – original draft, Formal Analysis, Project administration. GM: Validation, Visualization, Writing – review & editing. MG: Data curation, Formal Analysis, Methodology, Validation, Writing – review & editing. LB: Supervision, Validation, Visualization, Writing – review & editing. GC: Supervision, Validation, Visualization, Writing – review & editing. AR: Supervision, Validation, Visualization, Writing – review & editing. GP: Supervision, Validation, Visualization, Writing – review & editing. AB: Data curation, Formal Analysis, Investigation, Writing – review & editing. GT: Data curation, Supervision, Validation, Visualization, Writing – review & editing. CV: Data curation, Supervision, Validation, Visualization, Writing – review & editing. AC: Supervision, Validation, Visualization, Writing – review & editing. RM: Supervision, Validation, Visualization, Writing – review & editing. MAG: Supervision, Validation, Visualization, Writing – review & editing. LI: Supervision, Validation, Visualization, Writing – review & editing. LP: Conceptualization, Funding acquisition, Methodology, Project administration, Resources, Supervision, Validation, Visualization, Writing – review & editing.

## References

[1] Kerkmeijer LGW Valentini V Fuller CD(Slotman BJ. Editorial: online adaptive MR-guided radiotherapy. Front Oncol (2021) 11. doi: 10.3389/fonc.2021.748685 PMC8435672 34527596

[2] Kerkmeijer LGW Kishan AU Tree AC. Magnetic resonance imaging-guided adaptive radiotherapy for urological cancers: what urologists should know. Eur Urol (2022) 82(2):149–51. doi: 10.1016/j.eururo.2021.12.019 35031164

[3] Boldrini L Corradini S Gani C Henke L Hosni A Romano A. MR-guided radiotherapy for liver Malignancies. Front Oncol (2021) 11:616027. doi: 10.3389/fonc.2021.616027 33869001 PMC8047407

[4] Witt JS Rosenberg SA Bassetti MF. MRI-guided adaptive radiotherapy for liver tumours: visualising the future. Lancet Oncol (2020) 21(2):e74–82. doi: 10.1016/S1470-2045(20)30034-6 32007208

[5] Boldrini L Cusumano D Cellini F Azario L Mattiucci GC Valentini V. Online adaptive magnetic resonance guided radiotherapy for pancreatic cancer: state of the art, pearls and pitfalls. Radiat Oncol Lond Engl (2019) 14(1):71. doi: 10.1186/s13014-019-1275-3 PMC6489212 31036034

[6] McDermott LN Wendling M Sonke JJ van Herk M Mijnheer BJ. Anatomy changes in radiotherapy detected using portal imaging. Radiother Oncol (2006) 79(2):211–7. doi: 10.1016/j.radonc.2006.04.003 16698097

[7] Shortall J Vasquez Osorio E Chuter R Green A McWilliam A Kirkby K. Characterizing local dose perturbations due to gas cavities in magnetic resonance-guided radiotherapy. Med Phys (2020) 47(6):2484–94. doi: 10.1002/mp.14120 32144781

[8] Pham J Cao M Yoon SM Gao Y Kishan AU Yang Y. Dosimetric effects of air cavities for MRI-guided online Adaptive Radiation Therapy (MRgART) of prostate bed after radical prostatectomy. J Clin Med (2022) 11(2):364. doi: 10.3390/jcm11020364 35054061 PMC8780446

[9] Godoy Scripes P Subashi E Burleson S Liang J Romesser P Crane C. Impact of varying air cavity on planning dosimetry for rectum patients treated on a 1.5 T hybrid MR-linac system. J Appl Clin Med Phys (2020) 21(7):144–52. doi: 10.1002/acm2.12903 PMC7386179 32445292

[10] Uilkema S van der Heide U Sonke JJ Moreau M van Triest B Nijkamp J. A 1.5 T transverse magnetic field in radiotherapy of rectal cancer: Impact on the dose distribution. Med Phys (2015) 42(12):7182–9. doi: 10.1118/1.4936097 26632072

[11] Cusumano D Teodoli S Greco F Fidanzio A Boldrini L Massaccesi M. Experimental evaluation of the impact of low tesla transverse magnetic field on dose distribution in presence of tissue interfaces. Phys Med PM Int J Devoted Appl Phys Med Biol Off J Ital Assoc BioMed Phys AIFB (2018) 53:80–5. doi: 10.1016/j.ejmp.2018.08.007 30241758

[12] Shortall J Vasquez Osorio E Aitkenhead A Berresford J Agnew J Budgell G. Experimental verification the electron return effect around spherical air cavities for the MR-Linac using Monte Carlo calculation. Med Phys Jun (2020) 47(6):2506–15. doi: 10.1002/mp.14123 32145087

[13] Shortall J Osorio EV Cree A Song Y Dubec M Chuter R. Inter- and intra-fractional stability of rectal gas in pelvic cancer patients during MRIgRT. Med Phys (2021) 48(1):414–26. doi: 10.1002/mp.14586 33164217

[14] Jin H Kim DY Park JM Kang HC Chie EK An HJ. Dosimetric effects of air pocket during magnetic resonance-guided adaptive radiation therapy for pancreatic cancer. Prog Med Phys (2019) 30(4):104–11. doi: 10.14316/pmp.2019.30.4.104

[15] Su C Okamoto H Nishioka S Sakasai T Fujiyama D Miura Y. Dosimetric effect of the intestinal gas of online adaptive stereotactic body radiotherapy on target and critical organs without online electron density correction for pancreatic cancer. Br J Radiol (2021) 94(1119):20200239. doi: 10.1259/bjr.20200239 33353402 PMC8011255

[16] Estabrook NC Corn JB Ewing MM Cardenes HR Das IJ. Dosimetric impact of gastrointestinal air column in radiation treatment of pancreatic cancer. Br J Radiol (2017) 91:20170512. doi: 10.1259/bjr.20170512 29166133 PMC5965473

[17] Nardini M Placidi L. Chapter 6 - Robust online adaptive planning: Toward a uniform MR-LINAC treatment planning technique. In: Ozyar E Onal C Hackett SL, editors. Advances in Magnetic Resonance Technology and Applications. Cambridge, Massachusetts: Academic Press (2022). p. 101–22. Available at: https://www.sciencedirect.com/science/article/pii/B978032391689900025X. MR Linac Radiotherapy; vol. 8.

[18] Placidi L Nardini M Cusumano D Boldrini L Chiloiro G Romano A. VMAT-like plans for magnetic resonance guided radiotherapy: Addressing unmet needs. Phys Med (2021) 85:72–8. doi: 10.1016/j.ejmp.2021.05.002 33979726

[19] Kawrakow I Fippel M. Investigation of variance reduction techniques for Monte Carlo photon dose calculation using XVMC. Phys Med Biol (2000) 45(8):2163–83. doi: 10.1088/0031-9155/45/8/308 10958187

[20] Wang Y Mazur TR Green O Hu Y Li H Rodriguez V. A GPU-accelerated Monte Carlo dose calculation platform and its application toward validating an MRI-guided radiation therapy beam model. Med Phys (2016) 43(7):4040–52. doi: 10.1118/1.4953198 PMC4902823 27370123

[21] Miften M Olch A Mihailidis D Moran J Pawlicki T Molineu A. Tolerance limits and methodologies for IMRT measurement-based verification QA: Recommendations of AAPM Task Group No. 218. Med Phys (2018) 45(4):e53–83. doi: 10.1002/mp.12810 29443390

[22] Low DA Dempsey JF. Evaluation of the gamma dose distribution comparison method. Med Phys (2003) 30(9):2455–64. doi: 10.1118/1.1598711 14528967

[23] Parikh PJ Lee P Low DA Kim J Mittauer KE Bassetti MF. A multi-institutional phase 2 trial of ablative 5-fraction stereotactic magnetic resonance-guided on-table adaptive radiation therapy for borderline resectable and locally advanced pancreatic cancer. Int J Radiat Oncol Biol Phys (2023) 117(4):799–808. doi: 10.1016/j.ijrobp.2023.05.023 37210048

[24] Cusumano D Lenkowicz J Votta C Boldrini L Placidi L Catucci F. A deep learning approach to generate synthetic CT in low field MR-guided adaptive radiotherapy for abdominal and pelvic cases. Radiother Oncol J Eur Soc Ther Radiol Oncol (2020) 153:205–12. doi: 10.1016/j.radonc.2020.10.018 33075394

[25] Lenkowicz J Votta C Nardini M Quaranta F Catucci F Boldrini L. A deep learning approach to generate synthetic CT in low field MR-guided radiotherapy for lung cases. Radiother Oncol J Eur Soc Ther Radiol Oncol (2022) 176:31–8. doi: 10.1016/j.radonc.2022.08.028 36063982

